# Identification of Genes Required for Resistance to Peptidomimetic Antibiotics by Transposon Sequencing

**DOI:** 10.3389/fmicb.2020.01681

**Published:** 2020-07-23

**Authors:** Alessandra Vitale, Gabriella Pessi, Matthias Urfer, Hans H. Locher, Katja Zerbe, Daniel Obrecht, John A. Robinson, Leo Eberl

**Affiliations:** ^1^Department of Plant and Microbial Biology, University of Zurich, Zurich, Switzerland; ^2^Polyphor AG, Allschwil, Switzerland; ^3^Department of Chemistry, University of Zurich, Zurich, Switzerland

**Keywords:** transposon sequencing, antibiotic resistance, polymyxins, peptidomimetics, *Pseudomonas aeruginosa*

## Abstract

*Pseudomonas aeruginosa* is an opportunistic human pathogen and a leading cause of nosocomial infections. Due to its high intrinsic and adaptive resistance to antibiotics, infections caused by this organism are difficult to treat and new therapeutic options are urgently needed. Novel peptidomimetic antibiotics that target outer membrane (OM) proteins have shown great promise for the treatment of *P. aeruginosa* infections. Here, we have performed genome-wide mutant fitness profiling using transposon sequencing (Tn-Seq) to identify resistance determinants against the recently described peptidomimetics L27-11, compounds 3 and 4, as well as polymyxin B2 (PMB) and colistin (COL). We identified a set of 13 core genes that affected resistance to all tested antibiotics, many of which encode enzymes involved in the modification of the lipopolysaccharide (LPS) or control their expression. We also identified fitness determinants that are specific for antibiotics with similar structures that may indicate differences in their modes of action. These results provide new insights into resistance mechanisms against these peptide antibiotics, which will be important for future clinical development and efforts to further improve their potency.

## Introduction

Antimicrobial resistance is a global threat for the effective treatment of infections and is a major cause of mortality (Prestinaci et al., 2015). The rapid spread of antimicrobial resistance in bacteria is largely driven by antimicrobial exposure, often in connection with the misuse of antimicrobial compounds in health care and animal husbandry (Baquero et al., 1998; Chantziaras et al., 2014; Holmes et al., 2016). To restrict the spread of antimicrobial resistance and increased mortality rate, the World Health Organization developed a framework of regulations early in the 21st century (WHO, 2001). Yet the emergence of multidrug resistant pathogenic bacteria, including the so-called ESKAPE pathogens (*Enterococcus faecium*, *Staphylococcus aureus*, *Klebsiella pneumoniae, Acinetobacter baumannii*, *Pseudomonas aeruginosa*, and *Enterobacter* species) is particularly alarming (Rice, 2008). Among the ESKAPE pathogens, the Gram-negative bacterium *P. aeruginosa* is one of the leading sources of nosocomial infections associated with morbidity and mortality (Kang et al., 2003). *P. aeruginosa* is a facultative anaerobic Gram-negative bacterium, which is able to perform denitrification in the presence of nitrate or nitrite (Filiatrault et al., 2005; Williams et al., 2006; Palmer et al., 2007) and pyruvate/arginine fermentation when nitrate is absent (Eschbach et al., 2004; Schreiber et al., 2006). Due to its enormous metabolic versatility, this bacterium can adapt to a wide range of environments (Frimmersdorf et al., 2010).

The extensive use and misuse of antibiotics belonging to the polymyxin (POL) family [polymyxin B2 (PMB) and colistin (COL)], which constitutes the last therapeutic option for the treatment of some Gram-negative multidrug resistant infections, has led to the development of resistance to these antibiotics in different microorganisms (Rice, 2008; Carattoli et al., 2017; Xiong et al., 2017; Zhang et al., 2017). POLs are cationic peptides with a high affinity for negatively charged molecules such as the lipopolysaccharides (LPS) of the outer membrane (OM) of Gram-negative bacteria (Rabanal and Cajal, 2017). After binding to the LPS, the POLs displace the bridging divalent cations and initiate a process leading to permeabilization of the OM (Rabanal and Cajal, 2017). The lethal action of POLs is thought to be the result of the depolarizing effect on the cytoplasmic membrane (CM), but alternative mechanisms such as oxidative stress or interaction with OM proteins have also been suggested (Storm et al., 1977; van der Meijden and Robinson, 2015; Yu et al., 2015).

Polymyxin resistance is often associated with modifications to the LPS. In *P. aeruginosa* the two-component systems PmrAB, PhoPQ, ColRS, ParRS, and CprRS control expression of the LPS modification operon *arnBCADTEF*, responsible for the addition of 4-amino-4-deoxy-L-arabinose (L-Ara4N) to lipid A (McPhee et al., 2003; Olaitan et al., 2014). In addition to LPS modification, the multidrug efflux system MexXY–OprM was demonstrated to increase the fitness of the organism in the presence of POLs (Baron et al., 2016; Puja et al., 2020). Previous work has shown that a low concentration of magnesium (Mg^2+^), which is sensed *via* the PhoPQ and PmrAB systems (McPhee et al., 2006), induces expression of the *arn* operon and consequently leads to POL resistance (Brown and Melling, 1969; Brown and Watkins, 1970; Groisman et al., 1997). Mg^2+^ limitation not only triggers LPS modification but also induces quorum sensing, biofilm formation and the production of the virulence factors phenazine and pyochelin (Guina et al., 2003; Mulcahy and Lewenza, 2011).

**FIGURE 1:**
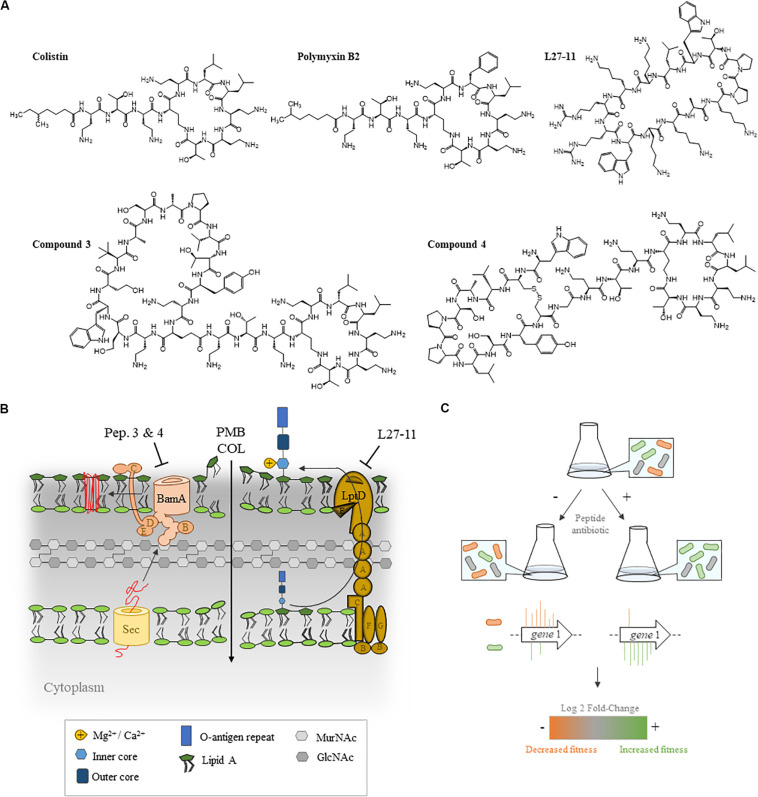
Experimental set-up of the transposon sequencing (Tn-Seq) analysis. **(A)** Structures of the five antibiotics employed in this study **(B)** and their cellular targets. Polymyxins (POLs) permeabilize the OM, leading to cytoplasmic membrane (CM) depolarization and cell lysis. Chimeras 3 and 4 bind to both lipopolysaccharide (LPS) and BamA, the main component of the β-barrel assembly machinery complex. L27-11 targets the OM protein LptD, which is involved in the transport of LPS to the cell surface. **(C)** A pool of *P. aeruginosa* UCBPP-PA14 transposon (Tn) insertion mutants was grown in the absence or presence of antibiotics and the number of Tn insertion sites were determined by Tn-Seq.

Novel cationic peptidomimetic antibiotics have recently been reported (Robinson, 2013) that show potent and selective action against *Pseudomonas* spp., including the opportunistic pathogen *P. aeruginosa* (Srinivas et al., 2010; Werneburg et al., 2012; Schmidt et al., 2013; Urfer et al., 2016). These antimicrobial compounds include L27-11 and the closely related clinical candidate murepavadin (also known as POL7080; Wach et al., 2018), which has a very low minimum inhibitory concentration (MIC_90_ 2 μg/mL) against *P. aeruginosa* (Ekkelenkamp et al., 2020). These compounds target the OM protein LptD, which is required for the transport of LPS to the OM (Srinivas et al., 2010; Werneburg et al., 2012; Schmidt et al., 2013). A more recently described family of chimeric peptides (referred to as chimeras), including compounds 3 and 4, show potent bactericidal activity against all the Gram-negative ESKAPE pathogens *E. coli*, *P. aeruginosa*, *A. baumanii*, and *K. pneumoniae*. These compounds bind to both LPS and the main component (BamA) of the β-barrel folding complex (BAM; Figures 1A,B) required for the folding and insertion of β-barrel proteins into the OM of Gram-negative bacteria (Luther et al., 2019). At present, these antimicrobial peptides are in preclinical development, however, knowledge about the mechanisms of resistance against these new classes of peptidomimetic antibiotics is scarce.

Several methods have been employed to identify intrinsic and adaptive resistance factors, including the isolation and characterization of resistant mutants (Fernández et al., 2013; Gutu et al., 2013) and the genome-wide analysis of gene expression of bacteria grown in the presence of sub-minimal inhibitory concentrations (sub-MICs) of the antibiotics (Cheah et al., 2016; Khaledi et al., 2016). The recently developed high-throughput transposon sequencing (Tn-Seq) technique has been demonstrated to be an alternative method to gain mechanistic insights into multidrug resistance mechanisms (Gallagher et al., 2011; Murray et al., 2015). Tn-Seq involves creating a saturated transposon (Tn) library of mutants, next-generation sequencing of the Tn insertion, and mapping the insertion sites to a reference genome. This technique has been widely used to identify genes that provide a fitness benefit for growth under a particular condition, as mutants with a growth defect would be depleted compared to a respective control culture (Murray et al., 2015; Solaimanpour et al., 2015; Rajagopal et al., 2016; Santiago et al., 2018).

In this study, we constructed a highly saturated Tn insertion library in the model organism *P. aeruginosa* University of California Berkeley Plant Pathology (UCBPP)-PA14 and used Tn-Seq to identify genes that provide a fitness benefit for growth in the presence of sub-MICs of PMB, COL and the novel peptidomimetic antibiotics L27-11, 3, and 4 (Figure 1C). Our data show that LPS modification influences susceptibility to all the antibiotics tested. However, we also identified distinct resistance determinants for each of the antibiotics, indicating differences in the mechanisms of resistance. Finally, we demonstrate that the production of spermidine promotes resistance against POLs and L27-11.

## Results

### Construction and Growth of a *P. aeruginosa* UCBPP-PA14 Transposon Library in the Presence of Sub-Lethal Concentrations of Different Peptide Antibiotics

A modified version of the suicide plasmid pLG99 (pLG99-Gm), which carries Tn*23* (Gallagher et al., 2013), was used to generate more than 500,000 insertion mutants in *P. aeruginosa* UCBPP-PA14. The Tn*5* derivative Tn*23* inserts randomly in the genome (Gallagher et al., 2013). In order to challenge the Tn mutants with the antibiotics, we first determined the MICs of PMB, COL and the peptidomimetic antibiotics L27-11, 4 and 3 in *P. aeruginosa* UCBPP-PA14 grown in basal medium 2 (BM2) supplemented with either high (2 mM) or low (20 μM) Mg^2+^ (Table 1). Previous work has shown that under Mg^2+^-limiting conditions *P. aeruginosa* induces expression of the *arn* gene cluster, which encodes enzymes that add an L-Ara4N moiety to the phosphate group of lipid A, thereby conferring resistance to POLs and other cationic antimicrobial peptides (McPhee et al., 2003, 2006). Accordingly, we determined that the MICs of PMB and COL for cells grown with 20 μM Mg^2+^ are at least 100-fold higher compared to cells grown in the presence of high Mg^2+^ (2 mM). In contrast to these POLs, we observed only a minor twofold change in susceptibility of *P. aeruginosa* UCBPP-PA14 to the peptidomimetic antibiotics 3 and 4 when grown with 20 μM Mg^2+^. The MIC for L27-11 was found to be approximately five times lower under limiting Mg^2+^ (20 μM) than under replete Mg^2+^ (2 mM) conditions. For the Tn-Seq experiments, we grew the Tn library of mutants in BM2 containing 20 μM Mg^2+^ for approximately 12 generations in the presence of sub-MICs of the different antibiotics that slowed down growth by 30% compared to the untreated control. The low Mg^2+^ concentration of the medium was used to induce resistance to the antimicrobial peptides (Table 1).

**TABLE 1:** Minimum inhibitory concentrations (MICs) of polymyxin B2 (PMB), colistin (COL), L27-11, 3 and 4 in basal medium 2 (BM2) in *Pseudomonas aeruginosa* UCBPP-PA14 wild-type.

	MIC (μg/ml)^a^		Sub-MIC(μg/ml) (L)^b^
	
Test Agent	BM2 (H)	BM2 (L)	
PMB	0.3	32	2
COL	0.15	64	2
L27-11	1	0.2	0.1
4	0.8	1.6	0.8
3	0.6	1.6	0.6

*^*a*^MICs were determined in BM2 containing replete (2 mM; High, H) or deplete (20 μM; Low, L) amounts of Mg^2+^ in 100 mL Erlenmeyer flasks. Data represent the mode values of three independent experiments. ^*b*^Sub-MICs of the antibiotics in BM2 containing deplete (20 μM; Low, L) amounts of Mg^2+^ were used for growth of the transposon (Tn) insertion library.*

### Identification of Fitness Determinants Involved in Susceptibility to Polymyxins, L27-11, and Peptides 3 and 4

After extraction of genomic DNA from the harvested Tn pools of each treatment, the Tn-Seq circle method (Gallagher et al., 2013) was employed to estimate the number of Tn insertion sites. Sequencing of the pools from the PMB and COL treatments resulted in 10.1 and 9.3 million reads, respectively, while the pools of the treatments with peptidomimetic antibiotics and the untreated controls resulted in 5.0–10.6 million reads (Supplementary Table S1). For all the samples, we were able to map more than 97% of the reads to the *P. aeruginosa* UCBPP-PA14 reference genome (Winsor et al., 2016). The number of unique insertion sites per gene was determined (Solaimanpour et al., 2015) and the insertion frequency of the Tn was calculated by dividing the size of the *P. aeruginosa* UCBPP-PA14 genome (6.5 Mbp) by the total number of unique insertion sites. We estimated that the Tn insertion sites are on average spaced by 14 bp in non-essential genes.

**FIGURE 2:**
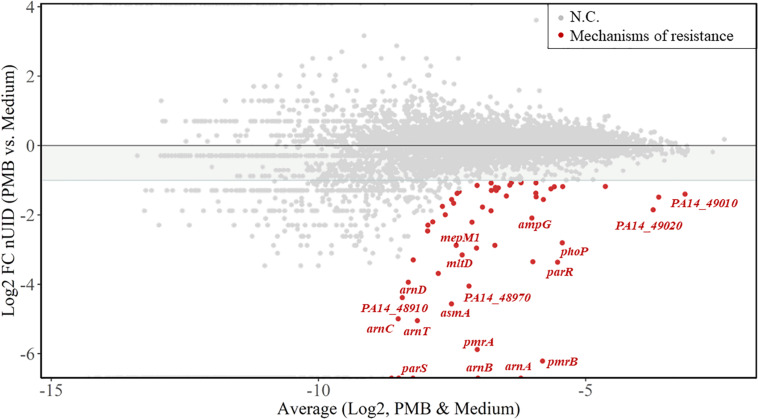
Resistance fitness determinants for polymyxin B2 (PMB). The MA-plot (M for log ratio and A for mean average) shows the average of the normalized unique insertion density (nUID) between the PMB-treated sample and the untreated sample (log2) on the *x*-axis and the fold change of the nUID between the two samples (log2) on the *y*-axis. The 58 genetic determinants likely involved in bacterial fitness upon PMB exposure are highlighted in red and genes of interest are named. NC, no change.

We next calculated the unique insertion density (UID) of each gene (Solaimanpour et al., 2015), which is the number of unique Tn insertions divided by the length of the gene. The UIDs were further normalized by the total number of unique insertions in each sample (normalized UIDs). The genes providing a fitness benefit for each of the treatments were determined by selecting a log2-fold change (based on normalized UIDs) of at least -1.0 between untreated and treated samples, as detailed in the section “Materials and Methods.” Figure 2 shows the log2-fold change of normalized UIDs of PMB-treated versus untreated samples plotted against the average normalized UIDs under both conditions. With the criteria employed, our analysis identified 58 fitness determinants following treatment with PMB, 42 for COL, 47 for L27-11, 75 for compound 4, and 43 for compound 3 (Supplementary Tables S2–S7). These genes were assigned to functional categories according to clusters of orthologous groups (Huerta-Cepas et al., 2017; Supplementary Figure S1).

### Polymyxins and Peptidomimetics Share Mechanisms of Resistance

**FIGURE 3:**
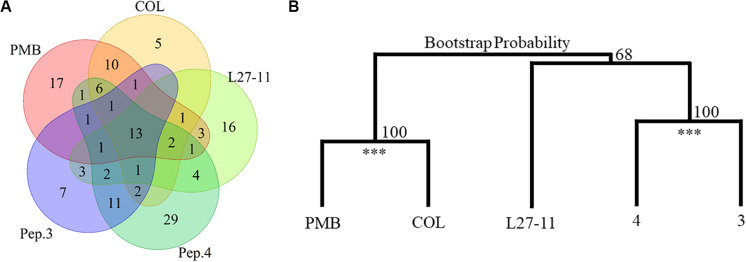
Number and clustering of fitness determinants. **(A)** The identified resistance determinants for the investigated antibiotics substantially overlap. Thirteen genes impact susceptibility to all tested antibiotics and besides these genes, PMB and colistin (COL) share 21 fitness determinants and peptides (Pep.) 3 and 4 have 19 common genetic determinants. **(B)** Cluster dendrogram of the Tn-Seq generated in response to PMB, COL, L27-11, peptides 3 and 4 treatments using the Ward criterion and the Euclidean distance. Peptides PMB/COL and 4/3 were clustered in 100% of the 100,000 iterations (Bootstrap Probability; ***p-value < 0.01).

Our Tn-Seq analysis identified 13 genes that had an impact on susceptibility to all peptide antibiotics tested (Figure 3A and Table 2). Many of these genes have previously been shown to affect resistance to PMB or COL, including five genes of the *arn* gene cluster (*PA14_18330* to *PA14_18370*), which directs the addition of L-Ara4N to lipid A. We also identified several genes of the regulatory systems that are known to be involved in the regulation of the *arn* gene cluster (Jeannot et al., 2017). Among these genes were the two-component systems *pmrAB* (*PA14_63150* and *PA14_63160*), *parRS* (*PA14_41260* and *PA14_41270*; except for treatment with peptide 3) and the response regulatory gene *phoP* of the PhoPQ system (PA14_49170 and PA14_49180). Another response regulatory gene that affects expression of the *arn* cluster, *colR* of the ColSR system (PA14_56940 and PA14_56950), showed a reduced number of Tn insertions only for the PMB treatment, while higher UIDs were observed for treatments with peptides 3 and 4 (Log2-fold change -1.0 versus + 1.29/1.15). In addition to the genes involved in LPS modification, *PA14_58090* encoding a hypothetical protein and two genes coding for bacteriophage Pf1 proteins (*PA14_48970* and *PA14_49000*; Kneale, 1983; Davis et al., 1995) provided a fitness benefit for all tested peptide antibiotics (Table 2). An involvement of the Pf1 phage in enhanced resistance to peptide antibiotics is further supported by the finding that various other Pf1 genes (*PA14_48900*, *PA14_48910*, *PA14_48940*, *PA14_48980*, *PA14_48990*, *PA14_49010*, and *PA14_49020*) were identified upon treatment with the different antibiotics (Supplementary Table S7). It has been demonstrated that the filamentous phages produced by *P. aeruginosa* cause the biofilm matrix to self-assemble into a liquid crystal that provides tolerance to aminoglycoside antibiotics (Secor et al., 2015). In the list of the 13 core genes, we also identified *PA14_42920* coding for the protein HsiJ2, which is predicted to be the basal plate component (TssK-2) of the second type VI secretion system (H2-T6SS; Lesic et al., 2009; Sana et al., 2012).

**TABLE 2:** Genetic determinants affecting susceptibility to all antimicrobial peptides tested (PMB, COL, L27-11, peptides 3 and 4).

Locus tag	Orthologs (PAO1)	Gene symbol	Description	Location (COG)^a^	PMB^b^	COL^b^	L27-11^b^	4^b^	3^b^
PA14_63150	PA4776	*pmrA*	Two-component response regulator	Cytoplasm (T)	−5.87	−4.86	−3.65	N/A	−4.00
PA14_63160	PA4777	*pmrB*	PmrB: two-component regulator system signal sensor kinase PmrB	CM (T)	−6.21	−7.19	−6.36	−6.25	−5.55
PA14_49180	PA1179	*phoP*	Two-component response regulator PhoP	Cytoplasm (T)	−2.80	−1.26	−4.05	N/A	N/A
PA14_18330	PA3556	*arnT*	4-amino-4-deoxy-L-arabinose transferase	CM (M)	−5.04	−5.03	−4.62	−4.56	−5.09
PA14_18340	PA3555	*arnD*	Hypothetical protein	Cytoplasm (G)	−3.93	−4.92	−4.62	N/A	N/A
PA14_18350	PA3554	*arnA*	Bifunctional UDP-glucuronic acid decarboxylase/UDP-4- amino-4-deoxy-L-arabinose formyltransferase	Cytoplasm (G,J,M)	N/A	N/A	−4.30	N/A	−7.58
PA14_18360	PA3553	*arnC*	Glycosyl transferase family protein	CM (M)	−4.99	−4.97	−3.96	N/A	N/A
PA14_18370	PA3552	*arnB*	UDP-4-amino-4-deoxy-L-arabinose–oxoglutarate aminotransferase	Cytoplasm (E)	N/A	−5.10	−3.80	−5.25	−5.78
PA14_58090	PA4476	*asmA*	Hypothetical protein	OM (S)	−4.56	−1.58	−1.16	−1.67	−1.68
PA14_42920	PA1667	*tssK-2*	HsiJ2	Cytoplasm (S)	−3.34	−3.47	−3.02	−3.07	−3.06
PA14_27190	–	–	tRNA-Ser	–	−1.87	N/A	−2.10	−1.94	−1.47
PA14_48970	PA0720	–	Helix destabilizing protein of bacteriophage Pf1	–	−4.04	−3.03	−2.42	−4.19	−1.91
PA14_49000	PA0717	–	Hypothetical protein	–	−1.48	−1.80	−1.67	−1.54	−1.28

*^*a*^COG, clusters of orthologous group; T, signal transduction; M, cell wall/membrane/envelope biogenesis; G, carbohydrate metabolism and transport; J, translation; E, amino acid metabolism and transport; S, function unknown; CM, cytoplasmic membrane; OM, outer membrane. ^*b*^Log2-fold change of normalized unique insertion densities (nUIDs) between the treated or untreated samples. N/A, not applicable as the fold change (FC) could not be calculated due to zero UIDs in the treated sample.*

To assess possible cross-resistances, we compared the fitness determinants required for growth in the presence of each of the peptides (Figure 3A). Besides the 13 core genes, the genetic determinants involved in PMB and COL susceptibility, two antibiotics with similar mechanisms of action (Kwa et al., 2007), overlap by 21 genes. Likewise, we observed an overlap of 19 genes between compounds 3 and 4, which target both LPS and BamA (Luther et al., 2019). In contrast, L27-11, which targets the OM protein LptD, showed fewer overlaps with the other antibiotics (9 with the POLs and 14 with peptides 3 and 4). These data suggest that the commonalities and differences between the sets of genes affecting susceptibility to the different antibiotics may be indicative of their diverse modes of action. This observation is further supported by a hierarchical clustering analysis of the Tn-Seq data, which revealed that PMB and COL and peptides 3 and 4 are clustered on two branches, while L27-11 forms a separate branch (Figure 3B).

### Resistance Factors Involved in Cell Envelope Biogenesis

A detailed analysis of our Tn-Seq data revealed that among the 21 fitness factors specifically affecting resistance to the two POLs were several genes affecting the structure and composition of the cell envelope (Supplementary Table S7). For example, we identified a gene involved in LPS biosynthesis, *PA14_23370*, which encodes an uridine diphosphate (UDP)-N-acetylglucosamine 2-epimerase (Burrows et al., 1996; Higgins et al., 2017). In agreement with our data, it has been shown that mutation of the orthologous gene *PA3148* in *P. aeruginosa* PAO1 increased OM permeability for the bovine neutrophil antimicrobial peptide indolicidin (Gooderham et al., 2008). Interestingly, more Tn insertions were observed in *PA14_23370* when the mutant library was treated with L27-11, relative to the untreated control, indicating an enrichment of this mutant in the presence of L27-11 (Supplementary Table S7). Among the core fitness genes is *PA14_58090* encoding an OM protein with a C-terminus homologous to AsmA, which was suggested to play a role in LPS biosynthesis in *E. coli* (Deng and Misra, 1996). While the exact function of AsmA is unclear, it has been speculated that AsmA plays a role in OM biogenesis by coordinating the assembly of OM proteins with the biosynthesis of LPS (Martorana et al., 2014). We also identified three genes potentially important for resistance to POLs and chimeras that encode proteins involved in the recycling of peptidoglycan (Sonnabend et al., 2020). The lytic transglycosylase SltB1 (PA14_12080) and the murein endopeptidase MepM1 (PA14_08540), which cleave peptidoglycan cross-connections, and the AmpG permease (PA14_57100), which imports peptidoglycan catabolites into the cytoplasm where they are degraded. To verify a role of AsmA and peptidoglycan turnover in antibiotic susceptibility, we tested respective Tn mutants in BM2 supplemented with 20 μM Mg^2+^. Mutants with defects in *asmA*, *mepM1*, *sltB1*, and *ampG* exhibited increased susceptibilities to POLs (2–16 fold). In contrast, with the exceptions of the twofold increased sensitivities of the *mepM1* and *asmA* mutants to the two chimeras and peptide 3, respectively, the mutations did not affect the sensitivity to the peptidomimetics (Table 3).

**TABLE 3:** Validation of fitness genes in *P. aeruginosa* UCBPP-PA14.

Locus tag	Description	Potential resistance	PMB^a^	COL^a^	L27-11^a^	4^a^	3^a^
PA14_08540	*mepM1*	PMB/COL/4	4	2	1	2	2
PA14_12080	*sltB1*	All (ex. L27-11)	4	2	1	1	1
PA14_57100	*ampG*	PMB/COL/4	4	4	1	1	1
PA14_58090	*asmA*	All	8	16	1	1	2
PA14_18370	*arnB*	All	64	32	8	8	2
PA14_63160	*pmrB*	All	16	8	4	2	2
PA14_49180	*phoP*	All	4	2	1	2	1
PA14_49170	*phoQ*	None	1	1	1	1	1
PA14_41260	*parR*	All (ex. 3)	4	16	1	1	1
PA14_56940	*colS*	None	2	2	2	1	1
PA14_63120	*speE*	L27-11	2	1	4	1	1

*^*a*^Fold-change decrease in MICs (increased susceptibility) relative to the wild-type (1 = MIC between wild-type and mutant is unchanged). Data represent the values of three independent experiments performed in BM2 containing 20 μM Mg^2+^. ex., except antibiotics shown.*

### The Role of LPS Modification in Peptide Antibiotic Susceptibility

To further explore the role of LPS modification in susceptibility, we determined the expression of the *arnB* and *pmrB* genes by qPCR in Mg^2+^ replete (2 mM) BM2 in the presence and absence of sub-MICs of POLs (0.1 μg/ml), L27-11 (0.4 μg/ml), peptides 3 (0.2 μg/ml) and 4 (0.3 μg/ml). The high Mg^2+^ concentration was chosen to inhibit LPS modification through the PhoPQ and PmrAB systems and to determine whether the antibiotics are capable of inducing LPS modification. Both genes were found to be up-regulated when the medium was supplemented with the antimicrobial peptides (Supplementary Table S8). A six to eightfold induction was observed with peptide 4, while the other antibiotics caused a two to threefold induction of *arnB* and *pmrB* expression. These data are in full agreement with previous work showing that various cationic peptides including PMB induce the *pmrAB* operon (McPhee et al., 2003) and suggest that peptide 4 is particularly efficient in inducing resistance.

We next tested Tn insertion mutants with defects in the biosynthesis or regulation of LPS modification for increased resistance against the peptide antibiotics in BM2 supplemented with 20 μM Mg^2+^. Strains with an inactivated LPS modification system (*arnB*) or defects in the regulators controlling expression of the *arn* gene cluster (*pmrB* and *parR*) exhibited markedly increased sensitivities to POLs (4–64 fold) and to a lesser degree (one to eightfold) to L27-11 and peptides 4 and 3 (Table 3). Previous work has shown that the PhoP-PhoQ system is highly expressed under limiting Mg^2+^ conditions and is involved in increased resistance to PMB *via* activation of the PmrA-PmrB system (Jeannot et al., 2017). Under Mg^2+^-limiting growth conditions, a *phoQ* mutant of *P. aeruginosa* PAO1 exhibited constitutive PMB resistance whereas a *phoP* mutant was unaffected, as in *P. aeruginosa* PhoP is not required for activation of LPS modification in low Mg^2+^ media (Macfarlane et al., 2000). Both the Tn-Seq data (Table 2) and the susceptibility testing of mutants (Table 3) are fully in line with these reports. Interestingly, the *phoP* mutant was found to be as susceptible to L27-11 and peptide 3 as the wild-type strain, despite the fact that the Tn-Seq analysis suggested a role of PhoP in increased resistance to these antibiotics. The ParR-ParS system can trigger upregulation of LPS modification in the presence of sub-MIC concentrations of POLs (Fernández et al., 2010). Mutants in *parR* and *parS* were shown to be highly sensitive to PMB and COL but not to other antimicrobial peptides (Fernández et al., 2010). Accordingly, we observed that a *parR* Tn insertion mutant exhibited increased sensitivity for POLs but none of the peptidomimetics (Table 3). Previous work has shown that deletion or disruption of the *colRS* genes, individually or jointly, abolishes the PMB resistance of a Δ*phoQ* mutant, but has no significant effect on susceptibility in a wild-type strain (Gutu et al., 2013). In line with these results, we only identified the *colR* gene in our Tn-Seq analysis, and a *colS* mutant displayed only a modest twofold increase in sensitivity for POLs and L27-11 (Table 3).

### Spermidine Protects Cells Against L27-11

We noticed that mutants in three genes (*PA14_63110* to *PA14_63130*), which are located immediately upstream of *pmrAB* were underrepresented in our Tn-Seq analysis of the L27-11 treatment (Supplementary Table S4). Gene *PA14_63130* was also identified as important for increased resistance under treatment with peptide 4. This gene cluster is involved in the biosynthesis of spermidine, which has been shown to protect bacterial membranes from oxidative stress and antibiotics, including PMB, possibly by competing for cation binding sites (Kwon and Lu, 2007; Johnson et al., 2012). To test the protective effect of spermidine against the peptide antibiotics, we determined the MICs of each antimicrobial compound in the wild type in the presence of spermidine. In agreement with our Tn-Seq analysis, we observed an eightfold increase in the MIC for L27-11 and a protective effect was also seen for PMB (Table 4). To further validate the role of spermidine for resistance to L27-11 and PMB, we tested a *PA14_63120* (*speE*) Tn mutant, which is unable to produce spermidine, for antibiotic susceptibility. The *speE* mutant exhibited increased susceptibilities to L27-11 and PMB (two to fourfold), confirming that spermidine can protect *P. aeruginosa* against these antibiotics (Table 3). Our data are in accordance with previous work that has shown that polyamines induce the expression of *phoPQ* and that the increased resistance to polymyxin B was abolished in a *phoP* but not in a *phoQ* mutant, suggesting that the PhoPQ system crosstalks with an unknown polyamine-specific signal transduction pathway in *P. aeruginosa* (Kwon and Lu, 2006, 2007). By contrast, exogenous spermidine increased the susceptibility of *P. aeruginosa* to the peptides 3 and 4. This is reminiscent of a previous report that showed that exogenous spermine and spermidine increased the MIC of imipenem against *P. aeruginosa* (Kwon and Lu, 2006, 2007), likely by blocking the OM porin OprD, which facilitates the penetration of imipenem through the OM (Hancock and Brinkman, 2002).

**TABLE 4:** Minimum inhibitory concentrations of the peptides (PMB, L27-11, chimeras 3 and 4) in the presence (4 mM) or absence of spermidine in *P. aeruginosa* UCBPP-PA14 wild-type.

Test Agent	Without Spermidine^a^	With 4 mM Spermidine^a^
L27-11	0.5	4
4	4	0.5
3	2	0.25
PMB	50	>100

*^*a*^MICs of the different antibiotics were determined in BM2 containing 20 μM Mg^2+^ in microtiter plates. Data represent mode values of three independent experiments.*

Collectively, these data suggest that the peptidomimetics have specific and complex interactions with the negatively charged cell envelope, perhaps through interactions with OM proteins such as BamA, a known target of these antibiotics (Luther et al., 2019).

### PMB Induces Interbacterial Competition

**FIGURE 4:**
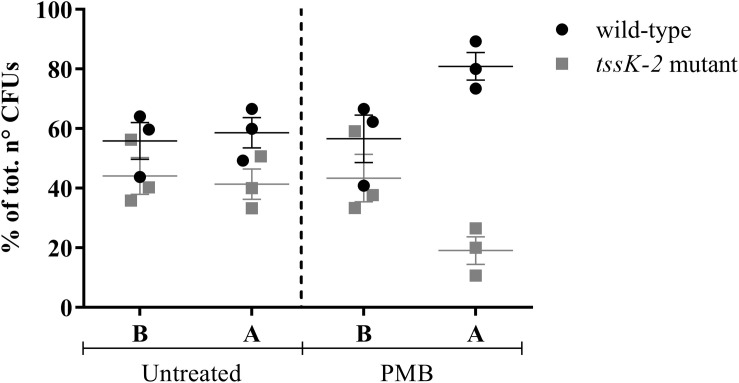
Competition between *Pseudomonas aeruginosa* UCBPP-PA14 wild-type and the mutant *tssK-2*. The strains were mixed (1:1; **B)** and grown until the end of the exponential phase **(A)** in 20 ml of basal medium 2 (BM2; 20 μM Mg^2+^) in the presence (2 μg/ml) or absence (untreated) of PMB. Data represent mean ± SEM of three independent experiments.

Confirming the results of the Tn-Seq analysis, all tested mutants showed increased sensitivity to the POLs (Table 3), except the *tssK-2* mutant, which exhibited wild-type levels of sensitivity to all tested antibiotics (data not shown). Previous studies revealed that the T6SS of *P. aeruginosa* is activated upon PMB treatment (Ho et al., 2013). In addition, expression of the orthologous gene in *P. aeruginosa* strain PAO1, *PA1667*, was shown to be induced by treatment with a sub-MIC of COL (Cummins et al., 2009). When a 1:1 mixture of the wild-type and a *tssK-2* Tn mutant was grown in BM2 containing 20 μM Mg^2+^ in the presence of a sub-lethal concentration of PMB, we observed that the *tssK-2* mutant was out-competed (Figure 4). Importantly, this effect was only seen in the presence of PMB. These data suggest that *tssK-2* has no direct function in antibiotic susceptibility but that the peptide antibiotics trigger bacterial T6SS-dependent competition. Additional work will be required to unravel the molecular mechanisms of TssK-2-mediated growth antagonism and to investigate whether other components of the second T6SS of *P. aeruginosa* UCBPP-PA14 are required for the function of TssK-2.

## Discussion

Recent research has identified novel antibiotics that target essential OM proteins (Ghequire et al., 2018; Storek et al., 2018a; Hart et al., 2019; Imai et al., 2019; Luther et al., 2019; Sousa, 2019). While these compounds show great promise for clinical applications, it is unclear how fast resistance against this new class of antibiotic may evolve and what the underlying mechanisms of resistance are. These will be important data for the clinical development of these antibiotics. In the case of the peptidomimetics L27-11 and murepavadin (POL7080), earlier studies linked a greatly increased resistance to both compounds to rare mutations in the target OM protein LptD (Srinivas et al., 2010; Werneburg et al., 2012). It is important to note, however, that our Tn-Seq analysis is unlikely to identify Tn insertions into essential genes, since these are mostly lethal. In addition, the mechanism of action of peptides 3 and 4 involves an interaction with BamA, an essential component of the BAM complex in the OM (Luther et al., 2019). Nevertheless, it is important to enquire whether mutations in other genes can affect the susceptibility of Gram-negative bacteria to these antibiotics. This is of particular importance in clinical development, where significant increases in MIC might influence the outcome of antibiotic treatment.

The aim of this study was, therefore, to identify genetic determinants in *P. aeruginosa* UCBPP-PA14 that confer a fitness benefit when exposed to the peptidomimetic L27-11, which targets the OM protein LptD (Srinivas et al., 2010; Werneburg et al., 2012) and the chimeras 3 and 4, which bind to BamA (Luther et al., 2019). To allow a detailed comparison, we have also included POLs in this study, which permeabilize the OM and are thought to depolarize the CM and cause cell lysis. Many studies have investigated the mechanisms of resistance to POLs in various Gram-negative bacteria using classical genetic approaches (Macfarlane et al., 2000; Fernández et al., 2010, 2013). More recently, Murray et al. (2015) employed a Tn-Seq approach to identify resistance mechanisms of *P. aeruginosa* PAO1 against PMB. However, this study only identified a single gene that encodes the hypothetical protein *PA14_66150*, but none of the well-characterized resistance determinants.

Here we identified a core set of 13 genes that impact upon various levels of susceptibility to all peptide antibiotics tested. Most of these genes encode enzymes for the addition of L-Ara4N to lipid A or are involved in the complex regulation of their expression. However, a more detailed analysis revealed important differences in the role of LPS modification in resistance to the tested peptide antibiotics. In accordance with previous studies (Groisman et al., 1997; McPhee et al., 2006), we observed that under low Mg^2+^ conditions (20 μM), which induces LPS modification, the MICs of the wild-type strain for PMB and COL is increased more than 100-fold relative to those under replete Mg^2+^ condition (2 mM; Table 1). In contrast, the MICs for peptides 3 and 4 only increased by a modest 2- and 2.5-fold, respectively, and in the case of compound L27-11 sensitivity was found to be even increased. To further evaluate the role of LPS modification, we determined the MICs of defined *arnB* (defective in LPS modification) and *pmrB* (deficient in the regulation of the *arn* biosynthetic cluster) mutants for the different antibiotics. Both mutants were found to be highly sensitive to PMB and COL (the MIC of the *arnB* mutant against PMB was decreased 64-fold) relative to the wild-type, whereas the MICs for the peptidomimetics 3, 4 and L27-11 were only decreased two to eightfold (Table 3). However, even the relatively small differences in susceptibility were sufficient to allow selection of mutants in the LPS modification pathway in our Tn-Seq analysis, indicating the high sensitivity of the experimental approach. Our data also support the findings of a recent study, in which strains resistant to POL7080 were isolated and shown to carry a mutation in the *pmrB* gene (Romano et al., 2019).

Our data demonstrate the important and intriguing trend that L-Ara4N modification of lipid A has only a moderate effect on susceptibility of *P. aeruginosa* to the peptidomimetics, although this lipid A modification is of major importance for resistance to the POLs in Gram-negative bacteria. This result might be explained by a different mechanism of action for the peptidomimetics, involving a specific binding to OM proteins. Interestingly, a mechanism of action involving an interaction with BamA has been proposed for lectin-like bacteriocins (LlpAs), which have been demonstrated to bind both BamA and LPS *via* their amino- and carboxy-terminal domains, respectively (Ghequire et al., 2018). In agreement with our results on compounds 3 and 4, the authors have shown that the absence of LPS binding is insufficient to cause full resistance to the LlpAs (Ghequire et al., 2018). In addition, the bactericidal activity of a recently described monoclonal antibody MAB1, which binds an epitope in the external loop L4 on BamA, was found to be linked to OM fluidity, which in turn is affected by LPS structure (Storek et al., 2018b). Hence, LPS modification may not only reduce the attraction of the antibiotics to the OM but could also impact membrane fluidity and thereby affect the antimicrobial activities of the compounds.

It is interesting to note that the three peptidomimetics are active against polymyxin-resistant strains (Sader et al., 2018; Luther et al., 2019), similar to what has been reported for some new generation polymyxins (Velkov et al., 2014). It is thought that these more hydrophobic polymyxin derivatives have increased potency, because they are insensitive to changes of the hydrophobic interior of the OM due to modification of lipid A acylation (Han et al., 2018). However, in the light of recent findings that some antibiotics bind specifically to essential OM proteins (Srinivas et al., 2010; Werneburg et al., 2012; Vetterli et al., 2018), it is possible that the novel polymyxin derivatives, in addition to their membrane-permeabilizing activities, also target membrane proteins. Additional work will be required to investigate this possibility. In addition to general resistance determinants, our Tn-Seq analysis also identified genes that provide a fitness benefit for only certain peptidomimetic antibiotics. This not only increases our understanding of the mode(s) of action but may also open new options for increasing the efficacy of these antibiotics.

## Materials and Methods

### Bacterial Strains and Growth Conditions

Bacterial strains, plasmids and primers used in this study are listed in Supplementary Table S9. Bacterial strains were routinely grown in lysogeny broth (LB; Miller, 1972) or BM2 (Fernández et al., 2012) supplemented with glucose as the sole carbon source. Media were supplemented with appropriate antibiotics using the following concentrations (in micrograms per milliliter): (i) for *Escherichia coli*, gentamicin (Gm) 10, kanamycin (Kan) 25, and trimethoprim (Tp) 50; (ii) for *P. aeruginosa*, Gm 20, nalidixic acid (Nal) 10.

### Tn-Seq Methodology

The pLG99 plasmid carries the Tn*5* derivative Tn*23*, which is composed of an outward-facing rhamnose-inducible promoter, a pMB1 replication origin and a *lacZ* gene, into which the gentamicin cassette has been inserted. To do so, the plasmid used for Tn delivery, pLG99 (Gallagher et al., 2013), was modified as follows: The gentamicin cassette of plasmid pBBR1MCS-5 (Kovach et al., 1995) was PCR amplified using the primers Gm_*Bcl*I_Fw and Gm_*Bcl*I_Rv. The amplicon was cleaned (QIAquick PCR Purification kit, Cat No./ID:28106), digested with *Bcl*I (New England Biolabs, Cat No. R0160S) and ligated into pLG99 cut with the same enzyme.

To construct a Tn library in *P. aeruginosa* UCBPP-PA14 (Rahme et al., 1995), 50 ml LB cultures of the donor strain *E. coli* CC118λ-pir/PLG99:Gm, the helper strain *E. coli* pRK2013 (Phadnis and Berg, 1987) and the recipient *P. aeruginosa* UCBPP-PA14 were harvested in the stationary phase, washed and resuspended in 6 ml 0.9% NaCl. Two hundred fifty microliters of the donor and the helper strains were mixed and incubated at room temperature for 10 min before 250 μl of the recipient strain was added. Next, 150 μl samples of the mix were inoculated onto 0.45 μM nitrocellulose membrane filters (Merck Millipore, Ref. HAQP02500), which were placed on LB agar plates. Following incubation at 37°C for 24 h, cells of nine filters were resuspended in 6 ml of 0.9% NaCl and 0.5 ml of this suspension was spread on 12 cm–2 square petri dishes containing LB agar supplemented with Gm 20 μg ml^–1^ and Nal 10 μg ml^–1^. After 20 – 24 h of incubation, the colonies were collected from the plates (417 square petri dishes in total) using 800 μl of LB supplemented with antibiotics. Afterward, 1.5 ml aliquots were amended with 50% glycerol and stored at -80°C.

To assess the concentrations of the peptides for challenging the library, the MICs of the peptides against *P. aeruginosa* UCBPP-PA14 were determined. An overnight culture of the strain was washed with minimal medium BM2 containing 20 μM Mg_2_SO_4_ (Fernández et al., 2012), the optical density (OD_600_) was adjusted to 0.2 and 1 ml of this suspension was added to 19 ml of BM2 supplemented with 20 μM Mg_2_SO_4_, 0.002% tween 80 (Sigma-Aldrich), 0.2% of Rhamnose (Sigma-Aldrich) and different concentrations of the peptide antibiotics (POLs: 8, 12, 16, 32, and 64 μg/ml; L27-11: 0.1, 0.2, and 0.4 μg/ml; chimeras: 0.4, 0.8, and 1.6 μg/ml; 100 mL Erlenmeyer). MICs were defined as no growth after 10 h, a time point when the non-treated culture reached the stationary phase. The library was grown overnight (starting OD_600_ = 0.01) in BM2 containing 20 μM Mg_2_SO_4_ to an OD_600_ of 0.6, harvested and grown (starting OD_600_ = 0.01) under the same conditions exponentially for six generations with and without the peptides. Cells were harvested (OD_600_ = 0.6), grown one more time under the same conditions for six generations before the cells were harvested and stored at -80°C.

### Sequencing of the Libraries Challenged With Peptide Antibiotics

Genomic DNA was extracted using the GenElute Bacterial Genomic DNA Kits (Cat. No. NA2100-1KT). The libraries were prepared using the Tn-Seq circle method (Gallagher et al., 2011) with the following modifications: the NEBNext Ultra II DNA Library Prep Kit for Illumina (Cat No. E7645S) was used for end repair and adaptor ligation. DNA fragments were separated on 1.5% agarose gels and the DNA was recovered using the QIAquick Gel Extraction Kit (Qiagen, Cat No./ID:28704). Amplification of the circularized DNA was done with the Phusion High-Fidelity DNA Polymerase (Thermo Fisher Scientific, Cat. No. F530L). Finally, the library was sequenced with the modified forward primer “T23_SEQ_G” and the reverse primer “PE_READ2_SEQ” using the MiSeq reagent kit (V2 300, cycles; Illumina, Ref. MS-102-2002) on the Illumina MiSeq platform. The adapters and the quality of the demultiplexed FASTQ reads were trimmed using the command line “cutadapt –a adapter –q quality –o output.fastq.gz input.fastq.gz” (Martin, 2011). The open source software Tn-Seq Explorer (Solaimanpour et al., 2015) was used to map the pair reads into the genome of *P. aeruginosa* UCBPP-PA14 (Winsor et al., 2016) and to count the number of Tn insertions using the default “–very-sensitive” command. The UID was determined with the same software by dividing the number of unique insertions in each gene per its length. The UID of each sample was normalized by the total number of unique insertion sites. The fold change (log2) was calculated by comparing the normalized UID (nUID) between the treated cells and the medium control, and this ratio was used to create a MA-plot, which shows the log2-fold change of the normalized UIDs on the *y*-axis and the average of the normalized UIDs between the PMB-treated sample and the untreated control on the *x*-axis. Each dot on the MA-plot refers to a gene and red dots depict fitness determinants. From each Tn-Seq data, our custom R-program selected the genes with a log2-fold change of at least -1.0 and a difference of at least 0.005 in the nUID between the untreated and the treated sample, which removed nUID smaller than 0.005 as low insertion counts may lead to false positive results (Wickham, 2017). The clusters of orthologous groups were assigned by using the online EggNOG 4.5.1 mapper (Huerta-Cepas et al., 2017) and Fisher tests were carried out online^1^ to assess potential over-representation of an EggNOG category. The R package “pvclust” was used in order to cluster the peptides according to the whole Tn-Seq data (nUID) with the Ward criterion and the Euclidean distance (Suzuki and Shimodaira, 2006) and a Venn diagram was constructed to compare the mechanisms of resistance (Dusa, 2018).

### Validation of the Tn-Seq Data

To validate the potential implication of some genes in the resistance to the peptidomimetic antimicrobial compounds, we looked for the respective mutant strains in the collection of single Tn insertion *P. aeruginosa* UCBPP-PA14 mutants (Liberati et al., 2006). The localization of Tn insertion was verified by PCR. After confirming the insertion mutants, the *arnB* mutant was grown in the absence and presence of the antibiotic in the same condition as the library was grown (*vide supra*). To test all the 11 strains in a high throughput way, the mutants were challenged with the peptide antibiotics in 200 μl BM2 containing 20 μM Mg^2+^ in 96-well-plates (Ref. 655161, Greiner bio-one) and the growth was recorded at the end of the exponential phase with the microplate reader “Infinite M200 PRO” (TECAN). To test the effect of spermidine (4 mM; Sigma-Aldrich) on the activity of the antimicrobial compounds, the standard twofold dilution MIC in 96-well-plates was performed (Srinivas et al., 2010).

### Quantitative Reverse Transcription PCR (qRT-PCR)

Ribonucleic acid (RNA) was extracted from early stationary cultures of the UCBPP-PA14 wild-type strain grown in BM2 containing 2 mM Mg_2_SO_4_ in the absence (control sample) or presence of antimicrobial peptides (approximately MIC_30_ corresponding to 0.1, 0.4, 0.3, and 0.2 μg/ml, for respectively, PMB, L27-11, 4 and 3). The RNA was further purified using the RNeasy Qiagen kit (Qiagen, Germany). First strand cDNA was synthesized using random primers (Invitrogen, United States) and Murine Leukemia Virus (MLV) reverse transcriptase (Promega, United States). qPCR was performed on the generated cDNA using Brilliant III Ultra-Fast SYBR^®^ Green QPCR Master Mix (Agilent, United States) and a Mx3000P instrument (Agilent, United States). Relative expression levels were calculated using the ΔΔ CT method (Pfaffl, 2001) and the *rpoD* gene (*PA14_07520*) was used as the reference gene for normalization.

## Data Availability Statement

The datasets generated in this study can be found in the NCBI short reads archive (SRA) platform in the Bioproject “*Pseudomonas aeruginosa* PA14 Tn-Seq (Peptide Antibiotics),” accession no. PRJNA562484 (https://www.ncbi. nlm.nih.gov/sra/?term=SRR11023345). Each sample can be found with the following accession numbers: BM2 dataset n°1, SRR11023345; BM2 PMB dataset, SRR11023344; BM2 COL dataset, SRR11023343; BM2 dataset n°2, SRR11023342; BM2 L27-11 dataset, SRR11023341; BM2 dataset n°3, SRR11023340; BM2 chimera 4 dataset, SRR11023339; and BM2 chimera 3 dataset, SRR11023338.

## Author Contributions

LE designed the study. AV constructed the Tn-Seq library and together with KZ, HL, and MU performed molecular genetics and microbiological studies. AV, MU, KZ, and GP collected and analyzed the data. JR and DO provided peptidomimetic antibiotics. AV, GP, JR, and LE wrote the manuscript, which was seen and agreed by all authors. All authors contributed to the interpretation of results.

## Conflict of Interest

HL, MU, and DO were employed by Polyphor AG. The remaining authors declare that the research was conducted in the absence of any commercial or financial relationships that could be construed as a potential conflict of interest.

## Footnotes

1 https://www.graphpad.com/quickcalcs/contingency1.cfm
