# Supplementary material for: Identification of Genes Required for Resistance to Peptidomimetic Antibiotics by Transposon Sequencing
Source: Front Microbiol. 2020 Jul 23;11:1681. doi: 10.3389/fmicb.2020.01681 (PMC7390954; doi:10.3389/fmicb.2020.01681)
Supplement: Supplementary file 1 [file Table_1.docx]

Supplementary Material

# Supplementary tables

**TABLE S1** Transposon sequencing results.

| **Growth condition** | **Total no. of reads^a^** | **No. (%) of mapped reads** | **No. of unique insertion sites** | **Insertion frequency (bp)** |
| --- | --- | --- | --- | --- |
| Untreated^b^ | 10,672,686 | 10,457,716 (97.98) | 448,753 | 15 |
| PMB | 10,112,421 | 9,992,513 (98.81) | 558,630 | 12 |
| COL | 9,389,544 | 9,258,016 (98.59) | 551,481 | 12 |
| Untreated^b^ | 7,359,113 | 7,219,581 (98.10) | 440,409 | 15 |
| L27-11 | 5,463,502 | 5,382,337 (98.51) | 474,405 | 14 |
| Untreated^b^ | 7,283,595 | 7,144,504 (98.09) | 440,216 | 15 |
| 4 | 5,097,450 | 5,046,269 (98.99) | 426,628 | 15 |
| 3 | 6,469,201 | 6,386,724 (98.72) | 623,751 | 11 |

^a^ The total no. of reads after trimming the quality.

^b^ The transposon library grown in the medium without antibiotic addition.

**TABLE S7** Genes that impact fitness of *P. aeruginosa* PA14 under different peptide treatments. Red backgrounds represent genes that were found depleted under conditions of peptides treatments when mutated (contributing to resistance) whereas green rectangles reveal genes with a higher fitness with a transposon insertion under antibacterial challenge. The numbers in cells represent the log2-fold change of the normalized UID between the treated and untreated samples. Bold numbers highlight the genes classified as fitness determinants (log2-fold change < - 1.0 and difference of normalized UID (between untreated and treated samples) > 0.005. FD, Fitness Determinants (the fold change could not be calculated because of zero values).


**TABLE S8** The effects the antibiotics on expression of *arnB* and *pmrB* was determined and the fold changes in expression calculated (N = 9, ± SD).

| **Locus tag** | **Description** | **Potential resistance** | **PMB** | **L27-11** | **4** | **3** |
| --- | --- | --- | --- | --- | --- | --- |
| PA14_18370 | *arnB* | All | 2.7±0.1 | 2.4±0.1 | 7.9±0.6 | 1.4±1.1 |
| PA14_63160 | *pmrB* | All | 2.3±0.2 | 2.6±0.1 | 5.6±0.2 | 2.3±1.5 |

**TABLE S9** List of bacterial strains, plasmids and oligonucleotides used in this study. Underlined oligonucleotides represent restriction enzyme.

| **Category** | **Description** | **Reference** |
| --- | --- | --- |
| **Strains** |  |  |
| ***P. aeruginosa*** |  |  |
| *P. aeruginosa* UCBPP-PA14 | Clinical isolate from burn wound, wild-type strain | (Rahme et al. 1995) |
| PA14-*arnB* | MAR2xT7::*PA14_18370* mutant of PA14 wt (*arnB*); Gm^R^ | (Liberati et al. 2006) |
| PA14-*pmrB* | MAR2xT7::*PA14_63160* mutant of PA14 wt (*pmrB*); Gm^R^ | (Liberati et al. 2006) |
| PA14-*phoP* | MAR2xT7::*PA14_49180* mutant of PA14 wt (*phoP*); Gm^R^ | (Liberati et al. 2006) |
| PA14-*phoQ* | MAR2xT7::*PA14_49170* mutant of PA14 wt (*phoQ*); Gm^R^ | (Liberati et al. 2006) |
| PA14-*parR* | MAR2xT7::*PA14_41260* mutant of PA14 wt (*parR*); Gm^R^ | (Liberati et al. 2006) |
| PA14-*colS* | MAR2xT7::*PA14_56940* mutant of PA14 wt (*colS*); Gm^R^ | (Liberati et al. 2006) |
| PA14-*tssk-2* | MAR2xT7::*PA14_42920* mutant of PA14 wt (*tssK-2*); Gm^R^ | (Liberati et al. 2006) |
| PA14-*asmA* | MAR2xT7::*PA14_58090* mutant of PA14 wt (*asmA*); Gm^R^ | (Liberati et al. 2006) |
| PA14-*ampG* | MAR2xT7::*PA14_57100* mutant of PA14 wt (*ampG*); Gm^R^ | (Liberati et al. 2006) |
| PA14-*mepM1* | MAR2xT7::*PA14_08540* mutant of PA14 wt (*mepM1*); Gm^R^ | (Liberati et al. 2006) |
| ***E. coli*** |  |  |
| GM2163 | wt strain; **genotype:** - dam-13::Tn9 (Camr) dcm-6 hsdR2 (rk-mk+) leuB6 hisG4 thi-1 araC14 lacY1 galK2 galT22 xylA5 mtl-1 rpsL136 (Strr) fhuA31 tsx-78 glnV44 mcrA mcrB1 | Fermentas |
| c118λ-pir | *Δ(ara-leu) araD ΔlacX74 galE galK phoA20 thi1 rpsE rpoB argE(Am) recAl* λ pir; Strep^R^ | (Herrero et al. 1990) |
| **Plasmids** |  |  |
| pLG99 | Transposon T23 (IS*lacZ*-P*_rhaB_* out-/FRT); Tp^R^ | (Gallagher et al. 2013) |
| pLG99::Gm | Transposon T23 (IS*lacZ*-P*_rhaB_* out-/FRT); Tp^R^, Gm^R^ | This study |
| pBBR1MCS-5 | Broad host-range cloning vector; Gm^R^ | (Kovach et al. 1995) |
| pRK2013 | Helper plasmid; Km^R^ | (Figurski and Helinski 1979) |
| **Oligonucleotides** |  |  |
| ***pLG99 ::Gm construction*** |  |  |
| Gm_BclI_Fw | tcctgatcaGACGCACACCGTGGAAA | This study |
| Gm_BclI_Rv | tcctgatcaGCGGCGTTGTGACAATTT | This study |
| pLG99_insert_Fw | AGGGCTGGTCTTCATCCACG | This study |
| ***Transposon-sequencing*** |  |  |
| PAIR_ADAPT_6BC##_HI | ACACTCTTTCCCTACACGACGCTCTTCCGATCTBBBBBBT | (Gallagher et al. 2013) |
| PAIR_ADAPT_6BC##_LO_5PH | BBBBBBAGATCGGAAGAGCGGTTCAGCAGGAATGCCGAG | (Gallagher et al. 2013) |
| T23_PAIR_COLLECT_1 | CTTCGGCGCGCCCTAGGGGGATCCTCGGCATTCCTGCTGAACCGCTCTTCCGATCT | (Gallagher et al. 2013) |
| T23_SLXA_PAIR_AmpF_3 | AATGATACGGCGACCACCGAGATCTACACTAGAGAATAGGAACTTCGGAATAGGAACTTCTTAGATGTGTATAAGAG | (Gallagher et al. 2013) |
| SLXA_PAIR_REV_AMP | CAAGCAGAAGACGGCATACGAGATCGGTCTCGGCATTCCTGCTGAACCGCTCTTCCGATCT | (Gallagher et al. 2013) |
| T23_SEQ_G | ATTAGGAACTTCGGAATAGGAACTTCTTAGATGTGTATAAGAGACAG | This study |
| T23_INDEX_1 | CTAGAGAATAGGAACTTCGGAATAGGAACTTCTTAGATGTGTATAAG | (Gallagher et al. 2013) |
| PE_READ2_SEQ | CGGTCTCGGCATTCCTGCTGAACCGCTCTTCCGATCT | (Gallagher et al. 2013) |
| ***Validation mutants /qRT-PCR*** |  |  |
| PA14_rpoD_Fw | GTTCATGCCGATCAAGCTG | This study |
| PA14_rpoD_Rv | ACGCAGAGTTGCATGATGG | This study |
| PA14_arnB_Fw | TGGATCTCGAAGGCCTGCG | This study |
| PA14_arnB_Rv | AGGTTCTTGATCGCGTGGA | This study |
| PA14_pmrB_Fw | CTTCACCCGCTTCTACCG | This study |
| PA14_pmrB_Rv | CCTGTACTTCCAGCCCTTTG | This study |
| PA14_phoP_Fw | CGCATCCTCGAATACCTCAT | This study |
| PA14_phoP_Rv | CAGCACCTCGATGACGTTG | This study |
| PA14_phoQ_Fw | GCCTACAGCATCGTCACCA | This study |
| PA14_phoQ_Rv | GTCAGACCTAGCCAGAGCAG | This study |
| PA14_parR_Fw | GTGGTGGTTCTCGACCTCAT | This study |
| PA14_parR_Rv | CGGCTTGATCACGTAGTCGT | This study |
| PA14_colS_Fw | GAGAACAGTCCTCCCACAGC | This study |
| PA14_colS_Rv | CTTGCTGCATACGGTGCAT | This study |
| PA14_tssk-2_Fw | CACCGGTAACCACATCGAG | This study |
| PA14_tssk-2_Rv | ACCTGCACCATGGAATCCT | This study |
| PA14_asmA_Fw | GACTGCTACGGATCGTCCTG | This study |
| PA14_asmA_Rv | AGTTGCTTGCCAGCTTCGT | This study |
| PA14_ampG_Fw | GACCATCACCCTCGACAACT | This study |
| PA14_ampG_Rv | AGCTGAGCATGGCGTACTG | This study |
| PA14_mepM1_Fw | CCTTCAGGAGAAAGACGACCT | This study |
| PA14_ mepM1_Rv | GGCTTGTCCGAGTCTTGCT | This study |
| PA14_speE_Fw | ATGGATACGCCGATCGAA | This study |
| PA14_speE_Rv | GTCGCTGTCCTCGATCGC | This study |
| PA14_sltB1_Fw | CCCAGGTTTGAGACCAAGAG | This study |
| PA14_s tB1_Rv | GTAGTCGCGGGTCATTTCG | This study |

# Supplementary figures

**
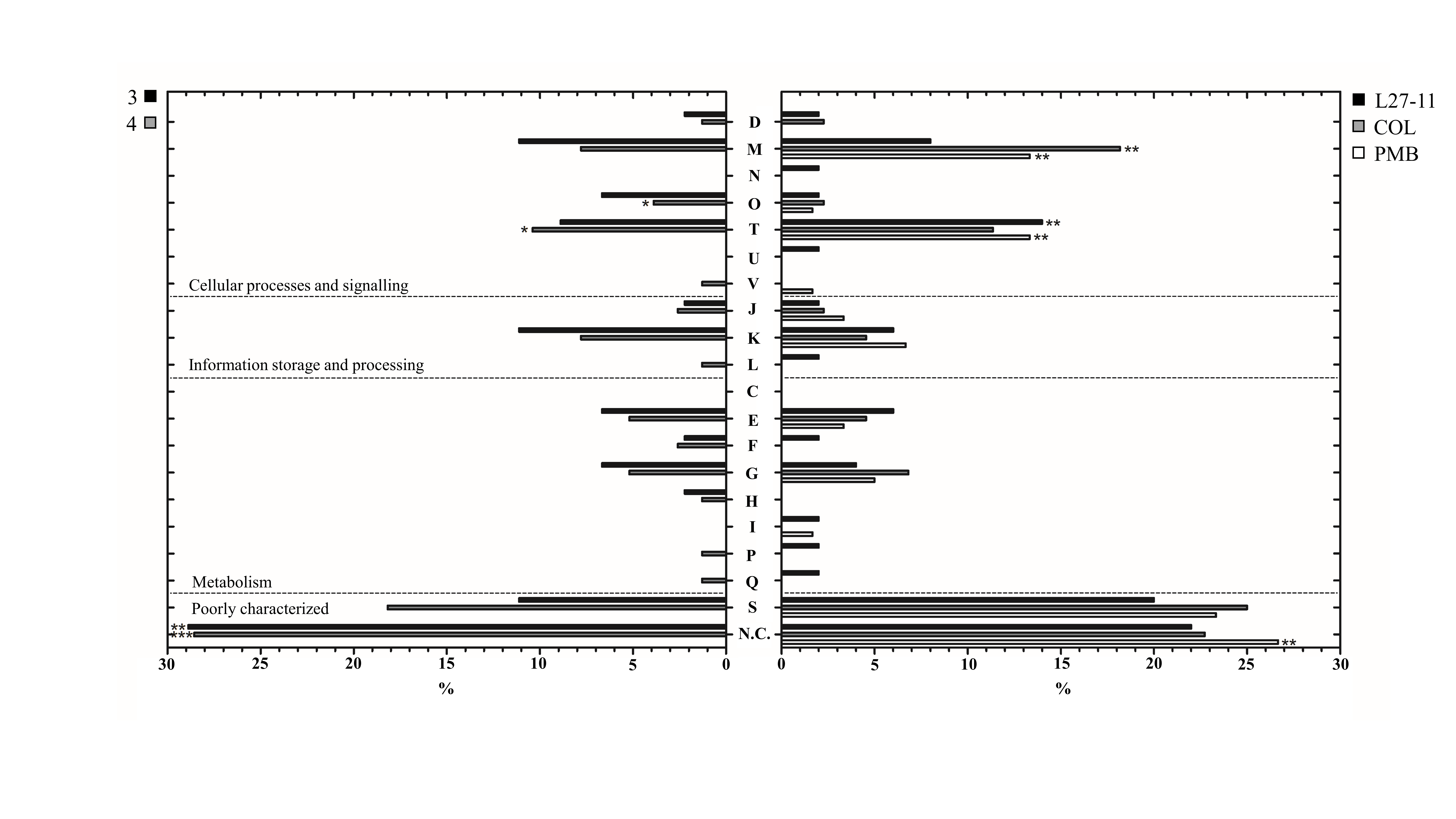
**

**FIGURE S1** Clusters of orthologous groups of the fitness determinants for PMB, COL, L27-11, 4 and 3. The clusters of orthologous groups of the fitness determinants were classified in four classes: poorly characterized; metabolism; information storage and processing; cellular processes and signalling. The percentage was calculated by dividing the number of genes in one category by the total number of genetic determinants for each peptide resistance. The asterisks indicate statistical significance (Fisher test, *p-*value < 0.05 (*); < 0.01 (**); < 0.001 (***)). K, Transcription; L, Replication, recombination and repair; D, Cell cycle control; M, Cell wall/membrane/envelope biogenesis; N, Cell Motility; O, Posttranslational modification, protein turnover, chaperones; T, Signal transduction mechanisms; U, Intracellular trafficking, secretion, and vesicular transport; V, Defense mechanisms; J, Translation, ribosomal structure and biogenesis; C, Energy production and conversion; E, Amino acid transport and metabolism; F, Nucleotide transport and metabolism; G, Carbohydrate transport and metabolism; H, Coenzyme transport and metabolism; I, Lipid transport and metabolism; P, Inorganic ion transport and metabolism; Q, Secondary metabolites biosynthesis, transport and catabolism; S, Function unknown; N.C., Not Categorized.

# References

Figurski, DH, and Helinski, DR. (1979). Replication of an origin-containing derivative of plasmid RK2 dependent on a plasmid function provided in *trans*. *Proceedings of the National Academy of Sciences of the United States of America* 76: 1648–52. doi: 10.1073/pnas.76.4.1648.

Gallagher, LA, Ramage, E, Patrapuvich, R, Weiss, E, Brittnacher, M, and Manoil, C. (2013). Sequence-defined transposon mutant library of *Burkholderia thailandensis*. *MBio* 4: e00604-13. doi: 10.1128/mbio.00604-13.

Herrero, M, Lorenzo, V De, and Timmis, KN. (1990). Transposon vectors containing non-antibiotic resistance selection markers for cloning and stable chromosomal insertion of foreign genes in Gram-negative bacteria. *Journal of Bacteriology* 172: 6557–67. doi: 10.1128/jb.172.11.6557-6567.1990.

Kovach, ME, Elzer, PH, Hill, DS, Robertson, GT, Farris, MA, Roop, RM, and Peterson, KM. (1995). Four new derivatives of the broad host range cloning vector PBBR1MCS, carrying different antibiotic resistance cassettes. *Gene* 166: 175–76. doi: 10.1016/0378-1119(95)00584-1.

Liberati, NT, Urbach, JM, Miyata, S, Lee, DG, Drenkard, E, Wu, G, Villanueva, J, Wei, T, and Ausubel, FM. (2006). An ordered, nonredundant library of *Pseudomonas* *aeruginosa* strain PA14 transposon insertion mutants. *Proceedings of the National Academy of Sciences of the United States of America* 103: 2833–38. doi: 10.1073/pnas.0511100103.

Rahme, LG, Stevens, EJ, Wolfort, SF, Shao, J, Tompkins, RG, and Ausubel, FM. (1995). Common virulence factors for bacterial pathogenicity in plants and animals. *Science* 268: 1899–1902. doi: 10.1126/science.7604262.
